# Retinal Organoids from Induced Pluripotent Stem Cells of Patients with Inherited Retinal Diseases: A Systematic Review

**DOI:** 10.1007/s12015-024-10802-7

**Published:** 2024-10-18

**Authors:** Yoo Jin Lee, Dong Hyun Jo

**Affiliations:** 1 Department of Medicine, Seoul National University College of Medicine, 103 Daehak-ro, Jongno-gu, Seoul, 03080 Republic of Korea; 2 Department of Anatomy and Cell Biology, Seoul National University College of Medicine, 103 Daehak-ro, Jongno-gu, Seoul, 03080 Republic of Korea

**Keywords:** Retinal organoid, Inherited retinal disease, Optic cup, Three-dimensional, Ophthalmology, Induced pluripotent stem cell

## Abstract

**Background:**

Currently, most inherited retinal diseases lack curative interventions, and available treatment modalities are constrained to symptomatic approaches. Retinal organoid technology has emerged as a method for treating inherited retinal diseases, with growing academic interest in recent years. The purpose of this review was to systematically organize the current protocols for generating retinal organoids using induced pluripotent stem cells from patients with inherited retinal disease and to investigate the application of retinal organoids in inherited retinal disease research.

**Methods:**

Data were collected from the PubMed, Scopus, and Web of Science databases using a keyword search. The main search term used was “retinal organoid,” accompanied by secondary keywords such as “optic cup,” “three-dimensional,” and “self-organizing.” The final search was conducted on October 2, 2024.

**Results:**

Of the 2,129 studies retrieved, 130 were included in the qualitative synthesis. The protocols for the generation of retinal organoids in inherited retinal disease research use five major approaches, categorized into 3D and a combination of 2D/3D approaches, implemented with modifications. Disease phenotypes have been successfully reproduced via the generation of retinal organoids from the induced pluripotent stem cells of individuals with inherited retinal diseases, facilitating the progression of research into novel therapeutic developments. Cells have been obtained from retinal organoids for cell therapy, and progress toward their potential integration into clinical practice is underway. Considering their potential applications, retinal organoid technology has shown promise across various domains.

**Conclusion:**

In this systematic review, we organized protocols for generating retinal organoids using induced pluripotent stem cells from patients with inherited retinal diseases. Retinal organoid technology has various applications including disease modeling, screening for novel therapies, and cell replacement therapy. Further advancements would make this technology a clinically significant tool for patients with inherited retinal diseases.

**Graphical Abstract:**

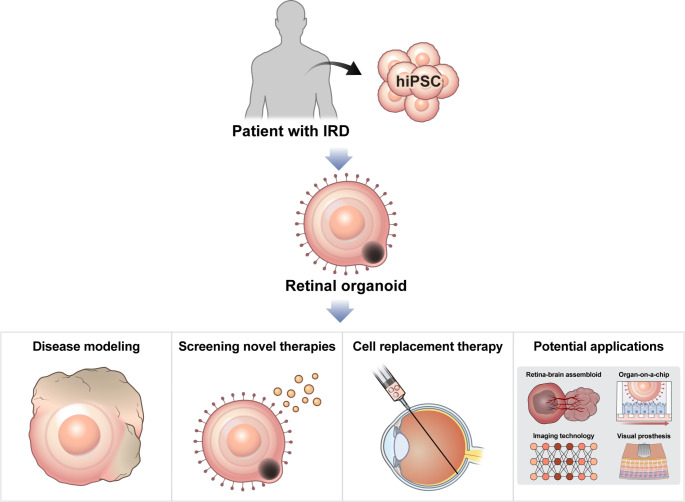

## Background

Inherited retinal disease (IRD) comprises a group of heterogeneous genetic disorders that cause blindness, affecting approximately 1 in 2,000 individuals worldwide [1, 2]. IRDs are characterized by the degeneration of photoreceptors and/or retinal pigment epithelium (RPE), resulting in a wide spectrum of clinical manifestations [3]. The genetic landscape of IRDs is highly complex, with over 260 genes contributing to the diverse spectrum of IRDs [4]. Retinitis pigmentosa (RP), the most common form of IRD, accounts for about 40% of cases, while other forms include Stargardt disease, Leber congenital amaurosis (LCA), and cone-rod dystrophies [5]. The inheritance patterns of IRDs exhibit significant heterogeneity, encompassing autosomal dominant, autosomal recessive, or X-linked modes, adding to the complexity of diagnosis and treatment [6]. Currently, most IRDs are incurable, and treatment options are limited to symptomatic treatment, which includes refractive correction, visual aids, and educational training [7]. Research to develop treatments for IRDs has relied on in vitro and in vivo models, such as retinal cells differentiated from pluripotent stem cells (PSCs) and murine models of retinal degeneration [8, 9]. However, the results from these models are often not well reproduced in patients, owing to the low resemblance of such models to the in vivo human retina [10]. This discrepancy highlights the need for more accurate and representative models of human retinal physiology and pathology [11].

Retinal organoid (RO) technology has undergone breakthroughs in IRD research over the last decade [12]. ROs are three-dimensional (3D) structures composed of retina-specific cell types, developed through the controlled differentiation of PSCs into organized retinal tissues [13]. ROs comprise all major retinal cell types, which are self-organized in a spatiotemporal pattern identical to that observed during in vivo retinal development [12]. Recapitulating the in vivo retinogenesis of the “forebrain-optic vesicle-optic cup,” retinal cells exhibit lamination similar to that observed in the native retina [14]. In addition, ROs have transcriptomic similarities with the in vivo retina and reproduce the phototransduction cascade and synaptic connections [15]. Particularly, ROs generated from patient-derived induced pluripotent stem cells (iPSCs) enable disease modeling, drug screening, and cell therapy in patient-specific genetic contexts [16]. Recently, RO technology has made further progress by integrating cutting-edge technologies such as organ-on-a-chip, microfluidics, and assembloid technology, which has enabled ROs to highly resemble the native retina, allowing scientists to study the physiology and pathophysiology of the developing human retina in unprecedented detail [14, 17].

Despite these advancements, the ongoing development of diverse RO protocols has led to a lack of consensus on an optimal approach [18]. As the importance of RO research in IRDs continues to grow, there is an increasing need to consolidate and analyze the available protocols [16]. While several reviews have addressed specific features of ROs, there remains a need for a comprehensive systematic analysis that specifically focuses on RO preparation protocols and their applications in IRD research. Therefore, in this review, we aim to systematically categorize and analyze diverse RO preparation protocols, explore ROs derived from iPSCs of patients with IRDs, and investigate the utility of ROs in identifying novel therapeutic strategies and cell therapies for IRDs. In addition, the review offers insights into potential future applications of ROs.

## Methods

### Search Strategy

We searched studies related to ROs using PubMed (U.S. National Library of Medicine, Bethesda, MD, USA), EMBASE (Elsevier Inc., Amsterdam, The Netherlands), and Web of Science (Clarivate Analytics, Philadelphia, PA, USA). The primary search keyword was “retinal organoid,” and the secondary keywords included “optic cup,” “three-dimensional,” and “self-organizing.” We included literature that comprised the protocols for the preparation of ROs, generation of ROs derived from iPSCs of patients, use of ROs for screening possible therapies, and application of ROs in cell replacement therapies. Studies that were relevant but did not include the search keywords were incorporated manually. The final search was conducted on October 2, 2024.

### Study Selection and data Extraction

The study mainly included original articles and reviews, and additionally referred to other types of literature, if relevant. The retrieved studies were classified based on the purpose of generating ROs. First, protocols for differentiating ROs were identified in each study. Then, the protocols were summarized and evaluated for procedural differences. The articles were then thoroughly studied for the following categorizations: patient disease mutation, type of therapeutic approach, and type of transplanted cell. In addition, potential applications suggested by the authors were reviewed.

Throughout the review process, two reviewers independently screened and cross-checked the literature from the databases. Disagreements were resolved by unanimous agreement between the reviewers and appraised by a third reviewer. The selected publications were organized and presented according to the Preferred Reporting Items for Systematic Reviews and Meta-Analyses (PRISMA) statement [19]. Meta-analysis was not performed due to the heterogeneity of methodologies and outcome measures across the included studies. A qualitative synthesis was conducted instead to comprehensively review the diverse approaches in RO research.

## Results

### Search Results

**Fig. 1:**
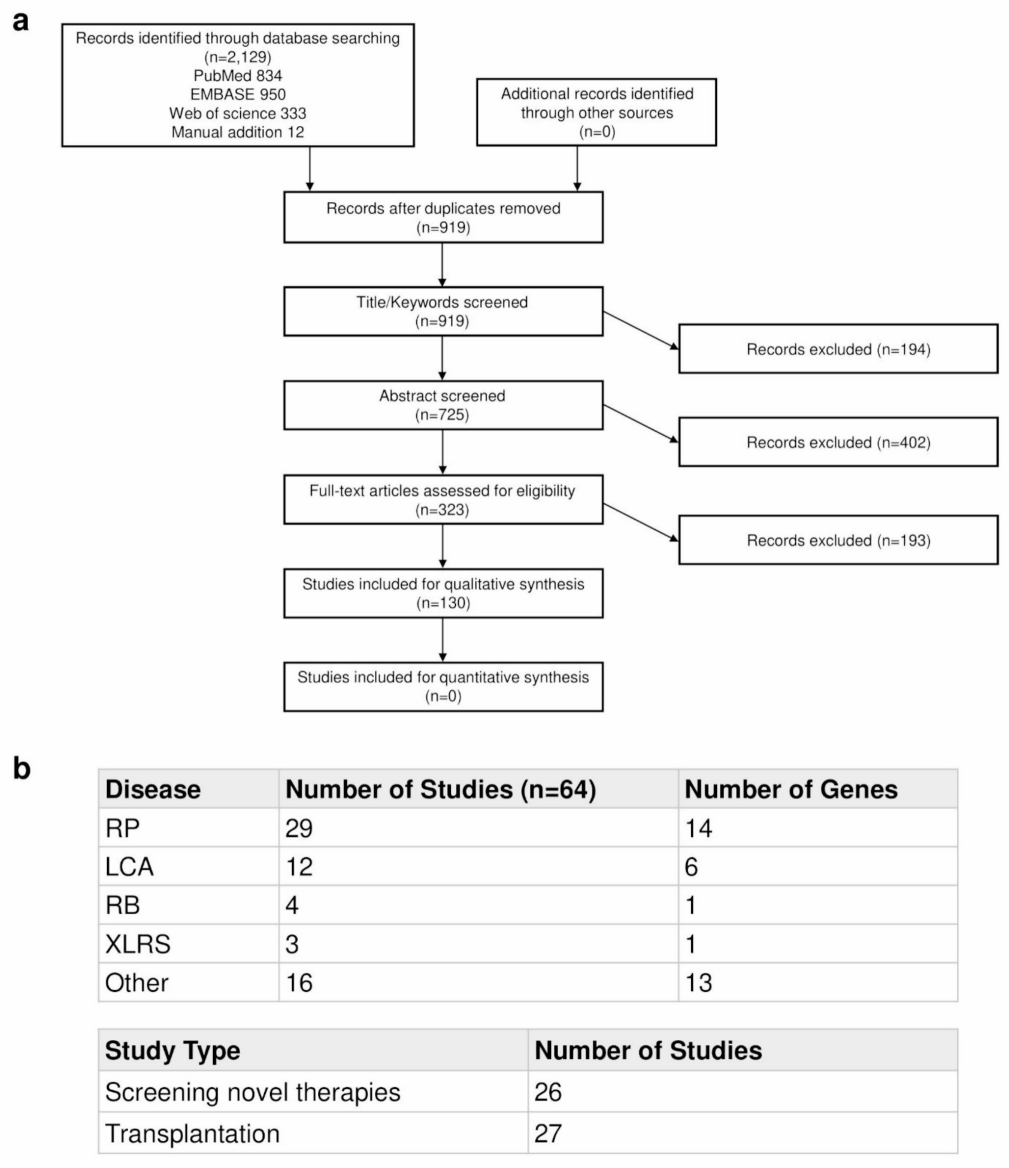
Summary of the search and selection process for studies on retinal organoids derived from patient induced pluripotent stem cells. (**a**) The flowchart illustrates the systematic search strategy, following the PRISMA guidelines [19]. Out of 2,129 identified studies, 130 were included in the qualitative analysis. (**b**) Our review encompasses 64 articles focused on differentiating retinal organoids from induced pluripotent stem cells of patients with inherited retinal diseases. These articles are categorized into 29 studies on retinitis pigmentosa, 12 on Leber congenital amaurosis, 4 on retinoblastoma, 3 on X-linked juvenile retinoschisis, and 16 on other inherited retinal diseases. Of these, 26 studies involved screening novel therapies using retinal organoids, while 27 focused on cell retrieval from retinal organoids for transplantation. Abbreviations: RP, retinitis pigmentosa; LCA, Leber congenital amaurosis; RB, retinoblastoma; XLRS, X-linked retinoschisis

A total of 2,129 studies were identified through the database search, and no additional records were identified from other sources. After removing duplicates, 919 records were subjected to title/keyword screening. Of these, 194 articles were excluded because of their low relevance to ROs. Abstract screening of the remaining 725 articles led to further full-text screening of 323 articles. Finally, 130 studies were subjected to qualitative screening following the PRISMA guidelines (Fig. 1a).

### Characteristics of the Included Articles

Our review included a total of 64 articles describing the differentiation of ROs from iPSCs derived from patients with IRDs (Fig. 1b). These 64 articles were categorized as follows: 29 studies on RP, 12 studies on LCA, 4 studies on retinoblastoma (RB), 3 studies on X-linked juvenile retinoschisis (XLRS), and 16 studies on other IRDs. Among these 64 articles, 26 studies described both the generation of ROs from patient-derived iPSCs and their use in screening therapeutic strategies, while 27 studies focused on the retrieval of cells from ROs for transplantation purposes.

### Protocols for Preparation of ROs from iPSCs Derived from Patients with IRDs

**Fig. 2:**
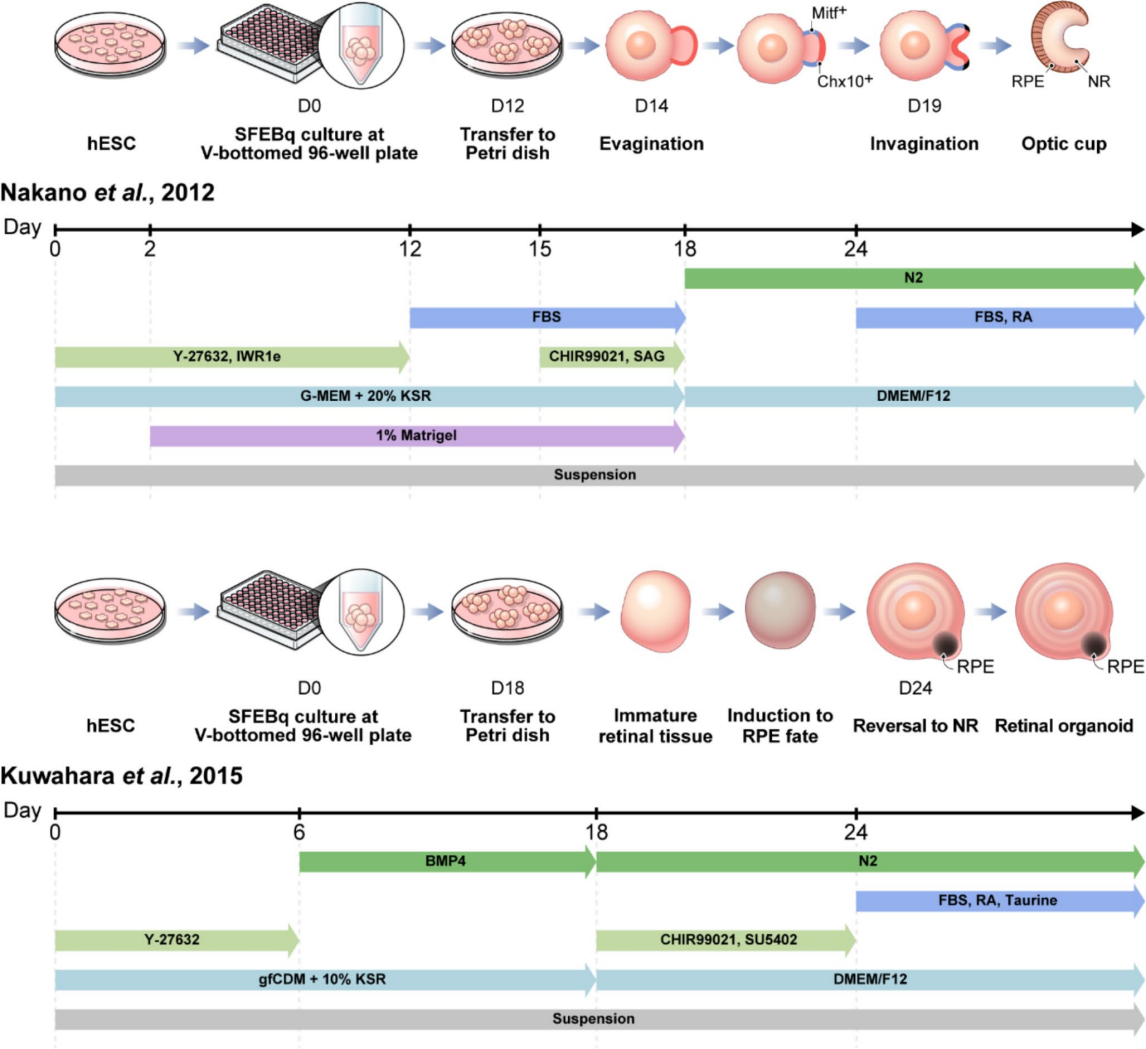
Schematic overview of 3D retinal organoid differentiation protocols. This figure illustrates two widely-cited 3D differentiation approaches for generating retinal organoids from human pluripotent stem cells. The protocol described by Nakano et al. [23] and the protocol by Kuwahara et al. [33] are presented, highlighting key stages such as embryoid body formation, differentiation, and retinal organoid maturation. The timelines indicate specific days for critical steps in each protocol. These methods have been successfully applied to patient-derived induced pluripotent stem cells for disease modeling, though researchers may modify these protocols based on specific research needs. Abbreviations: hESC, human embryonic stem cell; SFEBq, serum-free culture of embryoid body-like aggregates with quick reaggregation; RPE, retinal pigment epithelium; NR, neural retina; FBS, fetal bovine serum; RA, retinoic acid; SAG, smoothened agonist; KSR, knockout serum replacement; gfCDM, growth factor-free chemically defined medium

**Fig. 3:**
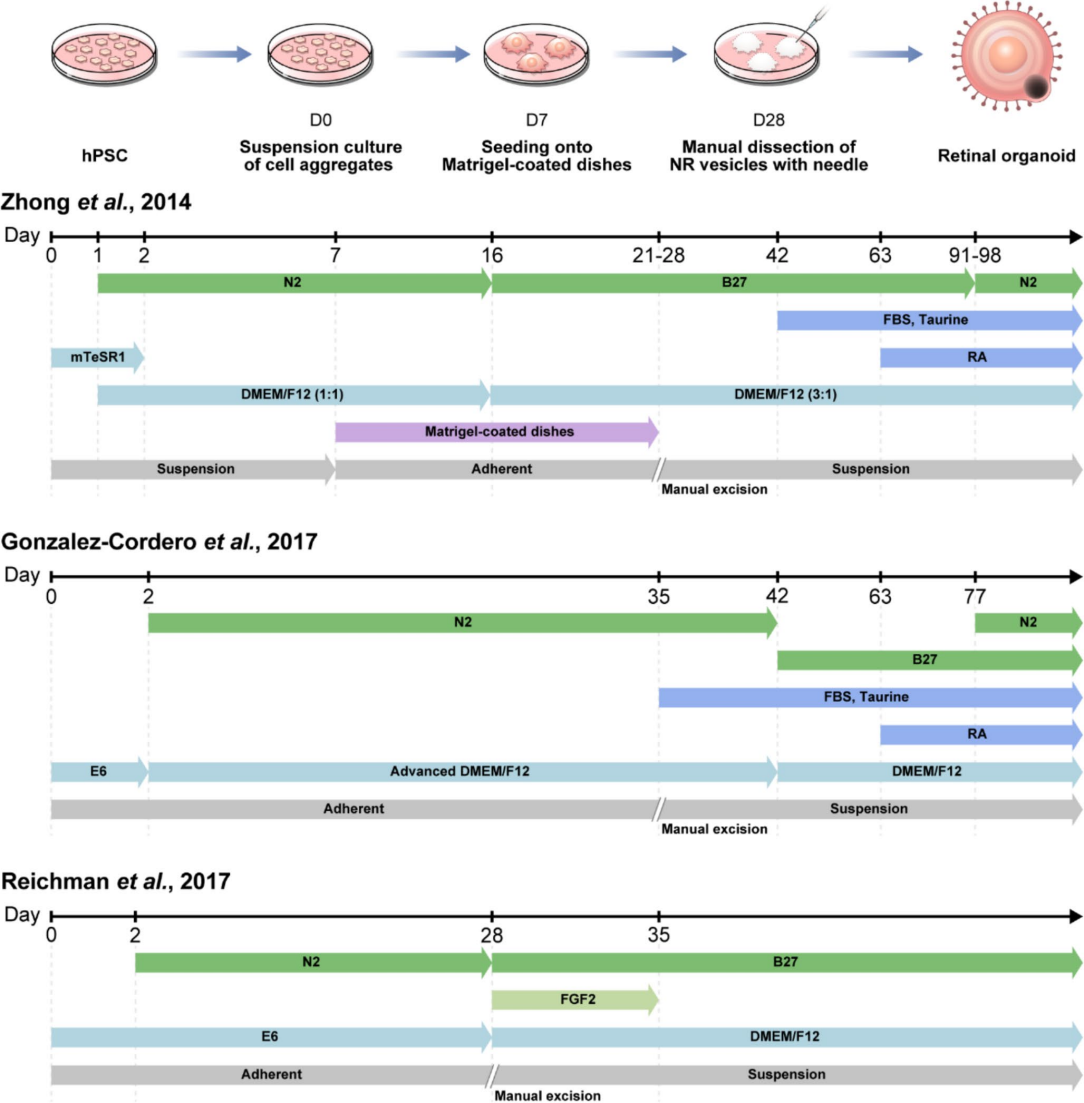
Schematic overview of 2D/3D retinal organoid differentiation protocols. This figure illustrates three widely-cited 2D/3D differentiation approaches for generating retinal organoids from human pluripotent stem cells. The protocols described by Zhong et al. [24], Gonzalez-Cordero et al. [41], and Reichman et al. [37] are presented, emphasizing the transition from 2D culture to 3D retinal organoid development. The timelines indicate specific days for critical steps in each protocol, providing a comparative view of different methodologies. These protocols have been successfully applied to patient-derived induced pluripotent stem cells for disease modeling, though researchers may modify these approaches based on specific research needs. Abbreviations: hPSC, human pluripotent stem cell; NR; neural retina; FBS, fetal bovine serum; RA, retinoic acid

Initial approaches for differentiating human PSCs (hPSCs) into photoreceptor cells were first described in 2006 [20, 21]. However, the overall efficiency of photoreceptor generation through these direct differentiation processes was low, falling short of the levels expected for clinical applications. Since then, several attempts have been made to replicate the in vivo developmental process of the optic vesicle-optic cup structure; these attempts were ultimately integrated by the Sasai lab, which pioneered the generation of self-organizing optic cup structures in 3D culture [22, 23]. Subsequently, RO studies focused on improving the 3D protocol, and, in 2014, took another major step by combining 2D and 3D cultures, thereby producing more mature ROs with functional photoreceptors [24]. Currently, five primary protocols are employed either directly or with slight modifications to generate ROs derived from patient-specific iPSCs (Table 1; Figs. 2 and 3) [23–27].

**Table 1:** Retinal organoids generated from patients with inherited retinal diseases

Disease	Gene	Mutation	Therapy screened	Protocol	References
RP	*CRB1*	c.1892A>G	−	[25]	[26]
		c.2548G>A			
	*CRB1*	c.3122T>C	AAV	[24]	[27]
		c.2983G>T			
		c.1892A>G			
		c.2843G>A			
		c.3122T>C			
	*CRB1*	c.1369C>T	−	[23]	[28]
		c.2027C>T			
	*CRB1*	c.2555T>C	−	[29]	[30]
		c.3014A>T			
	*CRB1*	c.3122T>C	−	[24]	[31]
		c.1892A>G			
		c.2911G>T			
		c.2843G>A			
		c.3122T>C			
	*CRB1*	c.2843G>A	−	[25]	[32]
		c.652+5G>C			
		c.2850_2855delAAATGC			
		c.4005+1G>A			
		c.2555T>C			
		c.3014A>T			
	*PRPF31*	c.1115_1125del11	CRISPR/Cas9	[33]	[34]
		c.522_527+10del			
	*PRPF31*	c.1205C>A	−	[29]	[35]
	*PRPF31*	c.1115_1125del11	−	[33]	[36]
		c.522_527+10del			
	*PRPF31*	c.709_734dup	AAV	[37]	[38]
		c.269_273del			
	*RPGR*	c.1685_1686delAT	CRISPR/Cas9	[33]	[39]
		c.2234_2235delGA,			
		c.2403_2404delAG			
	*RPGR*	Exon 14	CRISPR/Cas9	[33]	[40]
	*RPGR*	Frameshift mutations in exon ORF15	AAV	[41]	[42]
	*RPGR*	c.1685_1686delAT		[33]	[43]
		c.2234_2235delGA			
		c.2403_2404delAG			
	*RHO*	c.403C>T	−	[44]	[45]
	*RHO*	c.512C>T	−	[23]	[46]
	*RHO*	Structural variant	Small molecule	[24, 33]	[47]
	*USH2A*	c.2276G>T	CRISPR/Cas9	[37]	[48]
		c.2299delG			
	*USH2A*	c.8559-2A>G	−	[33]	[49]
		c.9127_9129delTCC			
	*USH2A*	c.8559-2A>G c.9127_9129delTCC	−	[33]	[50]
	*DYNC2H1*	c.12605_12606dup	−	[24]	[51]
		c.6632A>T			
		c.9836C>G			
		c.7987A>C			
		c.12716T>G			
	*IMPG2*	p.Tyr254Cys	−	[33]	[52]
		p.Ala805fs*			
	*MYO7A*	c.6070C>T	−	[41]	[53]
		c.223G>C			
		c.1996C>T			
		c.133-2A>G			
	*NR2E3*	c.166G>A	CRISPR/Cas9	[37]	[54]
	*PDE6B*	c.694G>A	−	[23]	[55]
	*RP17*	Structural variants	−	[41]	[56]
	*RP2*	c.358C>T	AAV	[23, 24]	[57]
	*TBC1D32*	c.317+5G>A	−	[37]	[58]
		c.846delTCCTA			
		c.18_27del			
		c.1141-1G>A			
		c.1267G>T			
		c.3513G>T			
	*TRNT1*	c.127_129delGAA	−	[23]	[59]
		c.1252delA			
		c.1252_1253insA			
		c.609-26T>C			
LCA	*CEP290*	c.2991+1655A>G	ASO	[23]	[60]
	*CEP290*	c.2991+1655A>G	ASO	[23]	[61]
	*CEP290*	c.2991+1655A>G	−	[24]	[62]
	*CEP290*	c.2991+1655A>G	Small molecule	[41]	[63]
	*CEP290*	−	Small molecule	[24]	[64]
	*AIPL1*	c.265T>C	−	[24]	[65]
	*AIPL1*	c.834G>A	TRID	[41]	[66]
		c.466-1G>C	CRISPR/Cas9		
		c.665G>A			
	*AIPL1*	c.834G>A	AAV	[41]	[67]
	*CRB1*	c.2548G>A	−	[37]	[68]
		c.4006-10A>G			
	*CRX*	c.264G>T	AAV	[24]	[69]
		c.413delT			
	*NPHP5*	c.421_422delTT	AAV	[24]	[70]
		c.1036G>T			
		c.1516_1517delCA			
	*RPE65*	c.200T>G	−	[24]	[71]
		c.430T>C			
RB	*RB1*	c.381-1G>A	−	[23]	[72]
		p.Glu137*			
		p.Asn258Glufs*10			
		p.Tyr318*			
		p.Glu323*			
		p.Leu343Leufs*5			
		p.Arg455*			
		p.Ile723Ser			
		p.Ile726fs			
		8.7 Mb del (13q14.11–q14.3)			
		Exon 1–17 deletion			
	*RB1*	c.2082delC	Small molecule	[33]	[73]
	*RB1*	c.623delT, c.958C>T	−	[33]	[74]
	*RB1*	c.2536C>T	−	[24]	[75]
		*RB1* gene deletion			
X-linked retinoschisis	*RS1*	c.625C>T	CRISPR/Cas9	[24]	[76]
		c.488G>A	Base editing		
	*RS1*	c.574C>T	−	[24, 33]	[77]
		c.365delA			
	*RS1*	c.214G>A	AAV	[22, 24]	[78]
Achromatopsia	*ATF6*	c.1699T>A	Small molecule	[24]	[79]
		c.970C>T			
	*ATF6*	c.1699T>A	−	[24]	[80]
Enhanced S-cone syndrome	*NR2E3*	c.119-2A>C	−	[23]	[81]
	*NR2E3*	c.119-2A>C	AAV	[24]	[82]
	*NRL*	c.223dupC	−	[33]	[83]
Stargardt disease	*ABCA4*	c.5196+1137G>A	ASO	[23]	[84]
*ABCA4*-associated retinal disease	*ABCA4*	c.4539+1321A>G	−	[23]	[85]
*CLCN2*-related retinal degeneration	*CLCN2*	c.2257C>T	−	[24]	[86]
Non- syndromic retinopathy	*CLN3*	c.175G>A	CRISPR/Cas9	[29]	[87]
		1 kb deletion			
Adult-onset rod-cone dystrophy	*CRB1*	c.1892A>G	−	[29]	[88]
		c.2548G>A			
X-linked rod-cone dystrophy	*RPGR*	c.1415-9A>G	−	[41]	[89]
Autosomal recessive rod-cone dystrophy	*UBAP1L*	c.910-7G>A	−	[37]	[90]
Autosomal recessive cone-rod dystrophy	*DRAM2*	c.140delG	−	[33]	[91]
		c.131G>A			
		c.494G>A			
Choroideremia	*CHM*	c.808C>T	−	[24, 33]	[92]
Early-onset pattern dystrophy	*OTX2*	c.259G>A	−	[33]	[93]
*EYS*-associated retinal dystrophy	*EYS*	c.8805C>A	HDAdV	[33]	[94]

The protocols referred to studies that had been used directly or with slight modifications. Abbreviations: *RP* retinitis pigmentosa, *LCA* Leber congenital amaurosis, *RB* retinoblastoma, *XLRS* X-linked retinoschisis, *AAV* adeno-associated virus-mediated gene augmentation, *ASO* antisense oligonucleotide, *TRID* translational readthrough-inducing drug, *HDAdV* helper-dependent adenoviral vector

Nakano et al. developed a method for creating stratified ROs using a serum-free culture of embryoid body-like aggregates with quick reaggregation (SFEBq) in suspension culture (Fig. 2) [23]. The protocol involved culturing aggregates in individual wells of a V-bottomed 96-well plate, followed by the sequential addition of various signaling cues: 20% knockout serum replacement, Matrigel, the Wnt inhibitor (IWR1e), Y-27,632, fetal bovine serum (FBS), and the smoothened agonist (SAG). This approach successfully steered PSCs into a retinal lineage. In addition, the application of the Wnt agonist (CHIR99021) at days 15–18 induced evagination of the retinal epithelium, resulting in the formation of an optic vesicle-like structure. Finally, invagination of the optic vesicle occurred at days 19–24, forming a double-walled optic cup structure; the inner layer became the neural retina (NR) with stratification of all major retinal cells, while the outer layer became the RPE.

To improve the 3D methodology, Kuwahara et al. introduced the “induction-reversal” approach, refining the process following the SFEBq stage (Fig. 2) [33]. Instead of utilizing NR induction medium (10% FBS, retinoic acid (RA), and taurine) after SFEBq, the aggregates were treated with the Wnt agonist (CHIR99021) and the FGFR inhibitor (SU5402) beginning on day 18 to induce RPE fate, followed by NR induction medium to reverse the process towards NR development. This strategy resulted in the generation of an RO with distinct NR and RPE domains, facilitating more efficient production of larger NR epithelia. Furthermore, the study demonstrated that precise temporal application of bone morphogenetic protein 4 (BMP4), specifically from day 6 to 18 of culture, enhanced the efficiency of NR epithelium formation. Notably, this approach eliminated the need for Matrigel, simplifying the differentiation process. Watari et al. developed the “induction-reversal method” by restructuring the process into five steps and refining the selection and preservation procedures for transplantation therapy [95].

The combination of 2D and 3D culture techniques by Zhong et al. involved the initial differentiation of iPSCs in a 2D culture, followed by a 3D suspension culture (Fig. 3) [24]. The process began with a neural induction medium comprising DMEM/F12 (1:1), N2 supplement, non-essential amino acids, and heparin. Subsequently, the medium was modified to DMEM/F12 (3:1) with B27 replacing N2, facilitating the formation of 3D retinal cups. At approximately week 4 of differentiation, manual dissection of the optic cup structure with adjacent RPE cells was performed. The dissected optic cup and RPE cells were then cultured in 3D suspension, with the RPE cells forming a clump next to the NR. At week 7, the medium was further supplemented with FBS and taurine, while RA was introduced at week 10 to support long-term culture. To promote photoreceptor maturation, the RA concentration was subsequently reduced, and B27 was replaced with N2 supplement. This protocol yielded ROs with functional photoreceptors exhibiting outer segment discs and light sensitivity. Although the method necessitated manual dissection, it demonstrated enhanced efficiency in RO generation compared to exclusively 3D approaches.

Following a similar approach to Zhong et al., both Gonzalez-Cordero et al. and Reichman et al. also developed 2D/3D protocols for RO generation, each introducing their own refinements and variations (Fig. 3) [37, 41]. Both Gonzalez-Cordero et al. and Reichman et al. initiated their protocols using E6 medium for early differentiation stages. Gonzalez-Cordero et al. conducted neural induction up to week 7 using proneural induction medium, consisting of DMEM/F12, non-essential amino acids, N2 supplement, and glutamine. Following manual excision, they utilized retinal differentiation medium (DMEM/F12, B27). The culture medium was enhanced with FBS, taurine, and GlutaMAX at week 6, with RA introduced at week 10. At week 12, the medium was further modified by the addition of N2 supplement, while the concentration of RA was decreased [41].

Reichman et al. further modified their approach by supplementing E6 medium with N2 after two days of culture. After manual excision on day 28, they transitioned to proB27 medium, which comprised DMEM/F12 (1:1), non-essential amino acids, and B27 supplement, with the addition of recombinant human FGF2. The FGF2 supplementation was discontinued after day 35 of culture [37]. Consequently, photoreceptors bearing connecting cilia with nascent outer segments were differentiated.

Subsequent studies have modified these self-organizing RO protocols to achieve higher efficiency in the generation of ROs. Timed introduction or elimination of certain signaling molecules enhanced RO production [96]. Kuwahara et al. developed an optimized culture method for feeder-free hPSCs by implementing three key strategies: preconditioning of the initial hPSC state through modulation of TGF-beta and Sonic hedgehog signaling pathways, application of SAG at day 0, and timed introduction of BMP4 [97]. Mellough et al. proposed a unique approach for introducing IGF-1 to induce laminated retinal tissue [25, 29]. Accelerated photoreceptor differentiation was achieved by 9-cis retinal, and, supplementation with BMP4 and IGF-1 was used to increase optic vesicle formation and lamination, respectively [98, 99]. RA, including all-trans RA, was used to encourage NR maturation [100, 101]; and timed Notch inhibition was used to induce photoreceptor fate specification [102]. Among cell types, cone-rich ROs have been produced without RA supplementation, while acceleration of ganglion cell development was achieved by encapsulating embryoid bodies in 3D Matrigel [103, 104]. Furthermore, co-culture systems with the RPE, microglia, or choriocapillaris have been devised to appropriately model the retinal environment [17, 50, 105–107].

### Generation of ROs from iPSCs Derived from Patients with IRDs

**Fig. 4:**
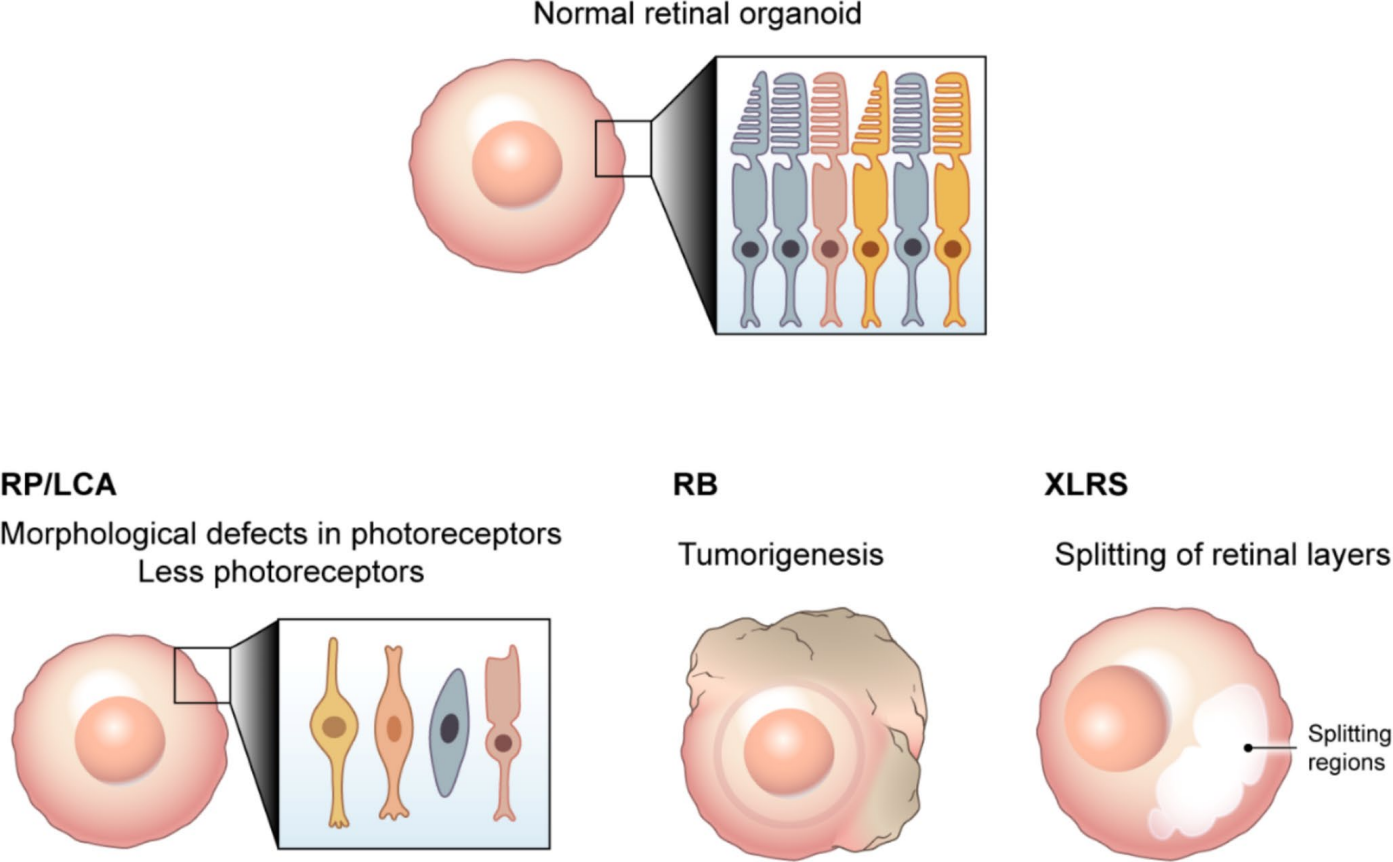
Morphological comparison of retinal organoids derived from healthy individuals and patients with inherited retinal diseases. Representative images show retinal organoids generated from induced pluripotent stem cells of healthy individuals and patients with retinitis pigmentosa, Leber congenital amaurosis, retinoblastoma, or X-linked retinoschisis. Abbreviations: RP, retinitis pigmentosa; LCA, Leber congenital amaurosis; RB, retinoblastoma; XLRS, X-linked retinoschisis

Retinal dystrophies representative of IRDs, such as RP, LCA, and RB, are discussed in the following subsections. Details of studies on ROs generated from patients with IRDs are presented in Table 1, and representative morphologies of ROs derived from healthy individuals and patients with IRDs are shown in Fig. 4, respectively.

#### Retinitis Pigmentosa

Retinitis pigmentosa (RP) comprises a genetically heterogeneous group of retinopathies characterized by the loss of photoreceptors and the presence of retinal pigment deposits, ultimately leading to blindness [108]. As the most common IRD, RP affects approximately 1 in 4000 individuals worldwide [56, 109]. Mutations in approximately 70 genes, including *RP2*, *RPGR*, *RHO*, *USH2A*, *CRB1*, and *PRPF31*, are known to cause RP [110].

Studies have successfully generated ROs from patient-derived iPSCs for 14 RP-associated genes, demonstrating the potential of this approach for disease modeling. These ROs generally exhibited proper lamination and contained identifiable photoreceptors and other retina-specific structures, while also manifesting structural abnormalities corresponding to individual genetic predispositions.

*USH2A* mutations, one of the most common causes of autosomal recessive RP, have been modeled using patient-derived ROs [111]. These models revealed early developmental abnormalities, including delayed self-organization, disrupted arrangement of retinal cells, abnormal retinal neuroepithelium differentiation, and defects in photoreceptor differentiation and cilium formation [48, 49]. These findings provide insights into the early pathogenesis of *USH2A*-associated RP.

ROs modeling *RPGR* mutations, primarily responsible for X-linked RP, demonstrated shortened cilia and defective photoreceptor development [39, 40]. *RPGR*-ROs exhibited increased expression of glial fibrillary acidic protein (GFAP), indicative of reactive gliosis, a phenomenon commonly observed in various forms of retinal degeneration [39, 42].

Patient-specific ROs with *RHO* mutations, a major cause of autosomal dominant RP, showed disrupted photoreceptor outer segment development and increased endoplasmic reticulum stress-induced apoptosis in rod cells, and gradual loss of rod photoreceptors [45, 46]. A recent study using ROs derived from patients with *RHO* copy number variations (CNVs) demonstrated photoreceptor dysgenesis, increased *RHO* mRNA expression, and elevated levels and mislocalization of rhodopsin protein within rod photoreceptors over an extended culture period [47].

Studies on *PRPF31* mutations using ROs and RPE cells revealed disrupted alternative splicing in genes crucial for splicing and ciliogenesis [34, 36]. *PRPF31*-mutant ROs showed splicing defects early in development, leading to subsequent photoreceptor degeneration.

ROs derived from patients with *CRB1* compound heterozygous mutations have been used to validate new iPSC lines [26, 28, 30]. A recent study on patient-derived *CRB1*-ROs demonstrated that a specific variant caused exon 11 skipping, thus providing evidence for the underlying pathogenic mechanism [32].

ROs with *NR2E3* mutations exhibited atypical rod photoreceptor development, characterized by a novel cell population expressing both rod and cone-specific genes, highlighting the gene’s critical function in photoreceptor fate determination and phototransduction pathway regulation [82]. Patient-derived *RP2*-ROs exhibited progressive rod photoreceptor degeneration, with peak cell death at day 150 and subsequent outer nuclear layer thinning by day 180, mirroring the clinical progression of X-linked RP [57]. *TBC1D32* and *DYNC2H1* variants both disrupted retinal ciliogenesis [51, 58]. *IMPG2*-mutant ROs showed photoreceptor outer segment abnormalities [52], while *MYO7A*-deficient ROs, associated with Usher syndrome type 1B, did not exhibit overt photoreceptor death [53]. *PDE6B*-mutant ROs recapitulated features of late-onset RP, including progressive photoreceptor degeneration [55]. *TRNT1*-associated RP ROs showed deficits in autophagy and inefficient expression of full-length TRNT1 protein [59].

Chronological events during defective retinogenesis have been observed in several models. For instance, *USH2A*-ROs showed early developmental abnormalities in the formation of the retinal neuroepithelium, preceding photoreceptor-specific defects [49]. In *PRPF31*-ROs, splicing defects were observed early in development, leading to subsequent photoreceptor degeneration [34].

These RO models have also been used to investigate obscure disease mechanisms. For example, Hi-C analysis of *RP17*-ROs demonstrated that structural variants can generate new topologically associating domains, leading to aberrant interactions between retinal enhancers and *GDPD1*, thus elucidating a potential gain-of-function mechanism in autosomal dominant RP [56]. Studies on *PRPF31*-associated RP using ROs have provided insights into the non-penetrance mechanism through dominant mutation carrier-derived ROs [35].

To validate their efficacy in representing the clinical phenotypes of RP as in vitro prototypes, these diverse RO models have been subjected to various experimental techniques. These include transcriptome analysis, immunofluorescence, western blotting, and morphological assessment, collectively providing a comprehensive approach for investigating the molecular and cellular basis of RP pathogenesis.

#### Leber Congenital Amaurosis

Leber congenital amaurosis (LCA) constitutes a family of congenital retinal dystrophies with serious clinical manifestations that cause vision loss during infancy [112]. It is the second most common IRD following RP, with a prevalence of 1 in 80,000 [113, 114]. Although not as common as RP, LCA is heterogeneous, with mutations in 26 genes known to cause LCA [110].

Five studies [60–64], comprising approximately 42% of the total studies, focused on generating ROs from iPSCs of patients with *CEP290*-associated LCA, the most common genetic cause of LCA. These studies successfully developed patient-specific optic cups with proper lamination, containing photoreceptors identified by specific markers. The ROs effectively recapitulated LCA pathogenesis, exhibiting defective ciliogenesis, with shorter and fewer cilia in photoreceptors compared to control ROs [60]. This finding was corroborated by observations of distinct cilia dysfunctions in *CEP290*-mutant ROs, including reduced cilia length and altered protein localization within the cilia [62]. These ciliary defects were consistent with the retinal degeneration and vision loss observed in patients with LCA, providing a potential mechanistic link between the cellular phenotype and clinical manifestations. Furthermore, *CEP290*-LCA ROs exhibited proteostasis imbalance, providing insights into the cellular mechanisms underlying the disease [64]. Collectively, these studies highlight the utility of *CEP290*-LCA ROs in modeling the cellular and molecular aspects of LCA, particularly in recapitulating the ciliary defects characteristic of the disease.

*AIPL1*-associated LCA-ROs closely resemble healthy control ROs but exhibit key molecular alterations characteristic of *AIPL1*-LCA [65–67]. These include decreased expression and mislocalization of AIPL1 and its interacting protein PDE6 in photoreceptor inner segments, as well as elevated cyclic guanosine monophosphate (cGMP) levels due to the absence of PDE6. Notably, despite these changes, *AIPL1*-LCA ROs do not display overt photoreceptor degeneration, contrasting with the severe early-onset degeneration observed in patients. This discrepancy suggests that while *AIPL1*-LCA ROs accurately model certain aspects of LCA pathogenesis, they may require further development, such as prolonged culture or interaction with RPE, to fully recapitulate the degenerative phenotype. These findings highlight both the potential and limitations of ROs in modeling complex retinal diseases.

*RPE65*-LCA ROs, generated from urine cell-derived iPSCs, demonstrated differentiation capabilities comparable to those of healthy controls [71]. These organoids exhibited typical retinal ultrastructure, including the outer limiting membrane, and photoreceptors with rudimentary outer segments in long-term culture, suggesting the potential of mutation-bearing iPSCs to differentiate into ROs and serve as disease models. *CRX*-LCA ROs exhibited reduced opsin expression in photoreceptors [69], whereas *NPHP5*-LCA ROs demonstrated impaired ciliogenesis, abnormal outer segment structures, and decreased CEP290 protein levels [70]. ROs from patients with LCA8 harboring *CRB1* mutations have been used to characterize the pathophysiology and disease phenotypes [68].

#### Retinoblastoma

Retinoblastoma (RB) is the most common intraocular tumor occurring in children, with a global incidence of 1 in 15,000–20,000 live births [115]. A mutation in the *RB1* gene, the translated protein of which is a key regulator of the cell cycle, is the sole cause of inherited retinoblastoma [116, 117]. Despite its relative genetic simplicity, the life-threatening aspects of retinoblastoma emphasize its clinical significance in IRD research.

RB-iPSC-ROs have been generated from patients and carriers of *RB1* germline mutations to assess the influence of patient context on tumor formation. The ROs displayed features of RB, rendering them an appropriate disease model [73]. ROs injected orthotopically into immunocompromised mice exhibited an in vivo RB formation indistinguishable from those of patient RBs [72]. Single-cell RNA sequencing (scRNA-seq) of RB-RO cells revealed retinal progenitor cells as the most common cell type and the co-expression of normally mutually exclusive genes such as *HES6* and *AIPL1* [72]. The results of sequencing could not distinguish RB-ROs from RBs of patients and orthotopic patient-derived xenografts in terms of cell type. In addition, a second hit mutation in patient iPSCs with a heterozygous mutation led to RB tumorigenesis in ROs, verifying Knudson’s “two-hit” hypothesis [74]. These outcomes imply that tumor tissues can be acquired without enucleation from multiple patients, and that RB can be derived from *RB1* mutation carriers who have never developed RB. In addition, ROs derived from iPSCs with heterozygous *RB1* mutations exhibited elevated levels of ATP and pyruvate, indicating significant alterations in energy metabolism without overt morphological changes during early retinal development [75]. These findings suggest that metabolic dysregulation may precede the onset of RB, highlighting the potential role of energy metabolism in the early stages of disease progression.

Several studies have reported the manipulation of healthy iPSCs and embryonic stem cells (ESCs) to retain *RB1* deletions using CRISPR/Cas9-mediated genome editing. Research on iPSC-RB-ROs contributed to the discovery that *RB1* deletion does not affect the maturation and proliferation of iPSCs, and that cell survival is unaffected despite the known function of *RB1* in inhibiting apoptosis [118]. Dysregulation of oncogenic pathways has been revealed via disease modeling using human ESC (hESC)-derived genetically engineered ROs [119, 120]. ROs have served as valuable models for studying RB, providing insights into the disease mechanisms [121, 122].

#### X-linked Juvenile Retinoschisis

X-linked juvenile retinoschisis (XLRS) is an IRD characterized by schisis (splitting) of the retina, primarily affecting males due to mutations in the *RS1* gene [76–78]. The *RS1* gene encodes retinoschisin, a protein crucial for maintaining retinal structural integrity and synaptic function.

ROs derived from patients with XLRS have been successfully generated, recapitulating key features of the disease and providing valuable insights into its pathophysiology. Interestingly, macroscopic morphological features of the diseased retina were also observed in ROs, further validating their relevance as disease models. These XLRS-ROs exhibited retinal splitting and defective retinoschisin production, mirroring the clinical manifestations of XLRS [76]. Molecular analyses of XLRS-ROs revealed dysregulation of Na/K-ATPase and increased ERK signaling pathway activity, potentially contributing to the disease mechanism [77]. Transcriptomic studies of XLRS-ROs demonstrated decreased expression of retinal cell markers, particularly those associated with photoreceptors, suggesting impaired retinal development [77, 78].

RNA sequencing of XLRS-ROs uncovered downregulation of genes involved in synapsis, nervous system development, and rhodopsin-like receptors, indicating delayed photoreceptor development [78]. Interestingly, co-culture experiments with control ROs during early differentiation stages showed partial recovery of the XLRS phenotype, highlighting the potential for cell-based therapeutic approaches [77].

#### Other Inherited Retinal Diseases

ROs are effective disease models for recapitulating retinal degeneration in various IRDs. Achromatopsia, an IRD caused by *ATF6* mutations, has been modeled using patient-derived ROs. These models failed to form regular cone photoreceptors, highlighting *ATF6*’s crucial role in human cone development [79]. A subsequent study investigated mitochondrial and endoplasmic reticulum function in achromatopsia-ROs, revealing dysfunctions that may contribute to the cellular pathology of this condition [80].

In Stargardt disease, a relatively common IRD with a prevalence of 1 in 8,000–10,000 [123], ROs derived from patient iPSCs with intronic *ABCA4* variants demonstrated splicing defects leading to retinal degeneration [84]. A study on *ABCA4*-associated retinal disease utilized ROs to investigate the c.4539+1321A>G variant, revealing its potential pathogenicity through mis-splicing in photoreceptor cells, resulting in a truncated protein. This variant was found to be potentially disease-causing when paired with a deleterious allele on the opposing chromosome [85].

ROs have also been valuable in studying less common IRDs. For instance, ROs modeling *CLN3*-associated non-syndromic retinopathy revealed novel transcripts inducing faulty CLN3 synthesis, toxic substance accumulation, and morphological defects in photoreceptor progenitor cells [87]. In a study of choroideremia using ROs, no significant differences were observed during early developmental stages. However, these ROs served as a valuable platform for validating isogenic iPSC lines, providing a foundation for future disease modeling studies [92].

Enhanced S-cone syndrome, caused by *NR2E3* or *NRL* mutations, has been effectively modeled using ROs [81–83]. scRNA-seq of these ROs revealed a unique branch point in the rod photoreceptor developmental lineage specific to the disease state. This study demonstrated *NR2E3*’s critical role in proper expression of phototransduction genes and identified regulatory sites influencing photoreceptor development [82].

In *DRAM2*-associated cone-rod dystrophy, ROs exhibited abnormalities in lipid metabolism, autophagic flux defects, and reduced lysosomal enzyme activity. These findings suggest *DRAM2*’s function in maintaining photoreceptor and RPE cell integrity through regulation of lysosomal function and autophagy [91]. A recent study established an iPSC line from a patient with early-onset pattern dystrophy carrying an *OTX2* c.259G>A variant, exploring the developmental capabilities of these mutation-bearing iPSCs in the context of retinal differentiation [93].

ROs can also function as a platform for evaluating the pathogenicity of certain mutations. In autosomal recessive rod-cone dystrophy, ROs facilitated the validation of in silico predictions regarding an *UBAP1L* intronic variant. RT-PCR experiments on 150-day-old ROs demonstrated that this variant leads to mis-splicing, likely resulting in the production of an elongated protein with altered function [90]. Similarly, ROs derived from patients with autosomal recessive adult-onset rod-cone dystrophy revealed the pathogenicity of two *CRB1* variants: c.1892A>G demonstrating exon skipping and c.2548G>A resulting in a missense mutation [88]. For X-linked rod-cone dystrophy, ROs have been employed to model *RPGR* mutations causing abnormal splicing [89]. In *EYS*-associated retinal dystrophy, RO studies uncovered the mislocalization of key proteins and heightened photoreceptor sensitivity to light stimuli, particularly blue light [94]. Interestingly, in the case of *CLCN2*-associated retinopathy, comparative analysis of patient-derived RPE and ROs indicated that RPE dysfunction, rather than photoreceptor degeneration, was the primary causative factor [86]. These diverse applications highlight the versatility of ROs in elucidating disease mechanisms and validating the pathogenicity of genetic variants across various IRDs.

Collectively, these findings underscore the ability of ROs derived from iPSCs of patients to facilitate a range of experiments using patient-derived tissues. This feature establishes a foundation for personalized medicine, in which the unique context of each patient is considered in the development of novel therapies. To maximize the utility of these models, researchers have increasingly employed advanced analytical techniques to delve deeper into the molecular and cellular dynamics within ROs. One such powerful tool is scRNA-seq, which allows for the high-resolution analysis of individual cellular profiles by capturing mRNA transcripts that reflect functional states [124]. This technology provides invaluable insights into the cellular heterogeneity of ROs by monitoring changes at a single-cell resolution, enabling the detection of subtle pathological changes and the evaluation of therapeutic interventions with unprecedented detail [53, 125]. By capturing the transcriptomes of individual cells at different stages of development, this technique provides a detailed view of cellular composition over time. Analyzing the transcripts of individual cells at the spatial and temporal levels provides insights into differentiation trajectories and allows for the identification of key signaling cues [126, 127]. Furthermore, scRNA-seq contributes to the enhancement of RO production protocols by offering novel perspectives on signaling pathways and cell type-specific markers [127].

### Use of Retinal Organoids for Screening Novel Therapies

**Fig. 5:**
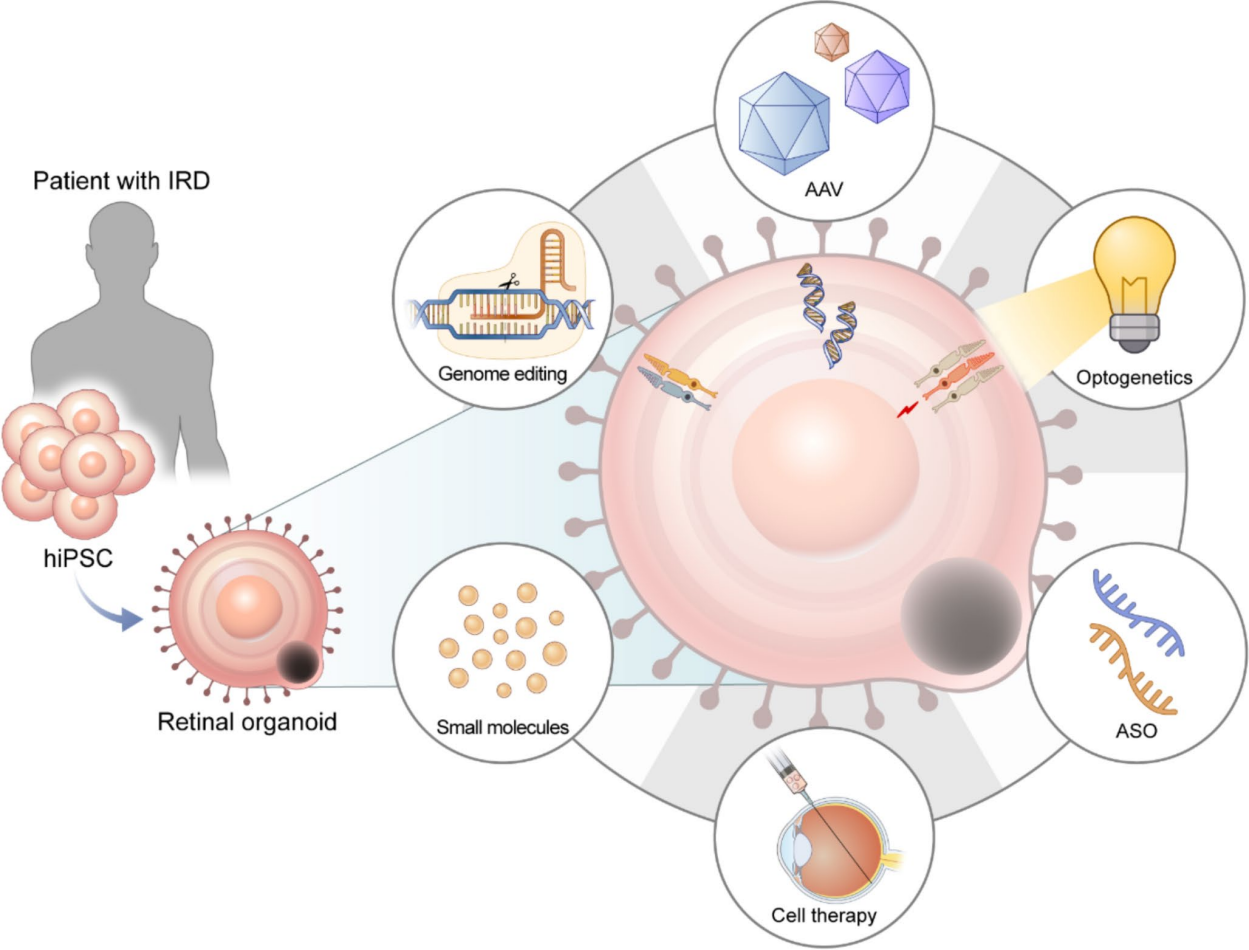
Therapeutic applications of retinal organoids derived from patients with inherited retinal diseases. Currently, retinal organoids are used for screening novel therapies such as genome editing, adeno-associated virus-mediated gene augmentation, optogenetics, antisense oligonucleotides, and small molecule treatments. Additionally, genetically-edited cells from patients can be used for autologous cell transplantation. Abbreviations: IRD, inherited retinal disease; hiPSC, human induced pluripotent stem cell; AAV, adeno-associated virus; ASO, antisense oligonucleotide

ROs have emerged as a valuable tool for investigating novel therapeutic strategies for IRDs (Fig. 5). Given that the majority of IRDs currently rely on symptomatic management, the development of targeted therapies addressing the fundamental disease mechanisms is of critical importance. Gene therapy has gained prominence in the field of IRD treatment, primarily due to the paucity of alternative therapeutic options. Both DNA- and RNA-based approaches have been explored in the context of gene therapy for IRDs.

#### DNA Therapy

Genome editing technologies, particularly the CRISPR/Cas9 system, offer the potential to directly modify pathogenic genes at the DNA level. This approach can effectively halt the production of mutant proteins in the edited cells by altering the underlying genetic sequence responsible for the disease phenotype. Genome-editing approaches using CRISPR/Cas9 have rescued the disease phenotype in iPSCs from patients with RP, XLRS, LCA4, LCA5, enhanced S-cone syndrome, or nonsyndromic retinopathy harboring *CLN3* mutations, by revealing differentiation into ROs [34, 38–40, 48, 54, 66, 76, 87, 128, 129]. In addition, an RPE-specific CRISPR/Cas9 system was directly applied to human ROs, demonstrating its potential for targeted cell therapy [130]. Furthermore, Cas9-directed base editing was tested on iPSCs derived from patients with XLRS, which confirmed the high repair efficiency of the base editors [76].

Alternatively, protein deficiencies resulting from genetic mutations can be addressed through the introduction of wild-type DNA sequences via adeno-associated virus (AAV) vectors into ROs. This approach, known as gene augmentation therapy, aims to restore normal protein expression and function in affected cells. RP phenotypes have been rescued by AAV-mediated *RPGR* and *CRB1* gene augmentation, respectively [42, 125]. In *AIPL1*-associated LCA4 ROs, AAV-mediated gene augmentation therapy effectively rescued molecular features, including the restoration of retinal PDE6 and reduction of elevated cGMP levels, with effects persisting for at least 70 days [67]. Furthermore, LCA-ROs ameliorated disease phenotypes via AAV-mediated *CRX* and *IQCB1*/*NPHP5* gene augmentation, respectively [69, 70]. ROs from *RP2* knockout iPSCs have been transduced with AAV2/5, which improved photoreceptor survival, thickened the outer nuclear layer, and restored rhodopsin expression, eventually reversing the RP phenotype [57]. AAV-driven *PRPF31* gene therapy could rescue photoreceptor cells in patient-derived ROs [38]. In XLRS-ROs, AAV2-mediated *RS1* gene augmentation partially restored delayed photoreceptor development and improved rod photoreceptor morphology [78]. For *EYS*-associated retinal dystrophy, a high-capacity helper-dependent adenoviral vector (HDAdV) was employed to deliver the full-length *EYS* gene, successfully rescuing protein mislocalization in organoids [94]. Notably, a study on enhanced S-cone syndrome ROs demonstrated that the timing of gene therapy is crucial, as most divergent rods became refractory to *NR2E3* supplementation by 130 days of differentiation, highlighting the importance of early intervention [82].

Optogenetics is an approach that introduces light-sensitive opsins, such as channelrhodopsins, into the eye so that the remaining retinal neurons can bear photosensitive properties. The technique can be especially applied for advanced-stage retinal degeneration in cases where other gene therapies are unavailable. Unlike genome editing or gene augmentation, which can only be utilized in patients with known genetic mutations, optogenetics is applicable regardless of the primary genetic defect. This is meaningful in IRDs, where genetic causes have been identified in only approximately 75% of cases [131]. To date, no studies have used patient-derived ROs to screen for optogenetic therapy, while normal human ROs have been used as screening tools to evaluate the membrane trafficking efficacy of microbial opsins [132]. Optogenetics in patient-derived ROs should be explored to evaluate the induction of opsins and the effectiveness of the therapy for further clinical applications, especially because transduction efficacy and photic responses vary depending on the condition of the cells. Eleven active/recruiting/completed clinical trials on the application of optogenetics in IRDs have been identified using the keyword “optogenetics” in ClinicalTrials.gov, with one showing encouraging results [133]. In this study, a patient with RP who underwent optogenetic therapy demonstrated partial visual recovery when using specialized light-transducing goggles. The patient exhibited improved ability to perceive, localize, count, and interact with various objects, representing a significant advancement in the field of optogenetic interventions for visual restoration.

#### RNA Therapy

Antisense oligonucleotides (ASOs) target pre-mRNAs or mRNAs to regulate their splicing and protein expression [134]. ROs derived from iPSCs of patients have been tested for several IRDs with ASO therapy. ROs derived from patients with Stargardt disease have been targeted for preclinical research on ASO-based medications to alleviate splicing defects in the *ABCA4* gene [84, 135]. Additionally, ROs derived from patients with *CEP290*-mutant LCA were treated with antisense morpholino (CEP290-MO) and ASO (QR-110) to reduce aberrant splicing and restore ciliogenesis [60, 61]. This treatment led to clinical trials with QR-110, in which patients showed vision improvements at three months, suggesting the potential of ROs as adequate pre-clinical research tools [136–138].

Nonsense suppression therapy using translational readthrough-inducing drugs (TRIDs) can also induce the production of full-length proteins by enhancing the insertion of cognate/near-cognate tRNAs. PTC124 (ataluren), the only TRID approved for clinical application, partially rescued nonsense mutations in *AIPL1*-LCA ROs [66]. Although their therapeutic effects are insufficient to reverse the disease phenotype, ROs have shown potential as testing platforms for novel drugs.

#### Small Molecules

Small-molecule drugs are reliable options for the treatment of IRDs. ROs derived from patients with achromatopsia were tested using a small-molecule ATF6 agonist (AA147). This small molecule reversed the transcriptional activity of the ATF6 variant, encouraging cone cell growth in ROs, suggesting a potential pharmacological approach [79]. In addition, RB organoids have been treated with chemotherapeutic agents (melphalan and topotecan), SYK inhibitors, and a small-molecule inhibitor of Bcl-2, resulting in decreased cell proliferation [73, 119]. ROs derived from patients with *CEP290* LCA10 demonstrated improved cilia formation and restored photoreceptor survival after treatment with eupatilin and resperine, respectively [63, 64]. A recent study demonstrated that Photoregulin3, a small molecule targeting NR2E3, partially rescued rhodopsin protein localization in ROs derived from patients with *RHO*-CNV-associated RP, suggesting a potential therapeutic approach for modifying disease progression [47].

Thus, patient-specific ROs have the potential to screen novel therapies or test existing drug combinations in individual patients. However, research on delivery modalities for these treatments is essential because therapeutic outcomes depend strongly on delivery efficacy. Notably, AAV is a promising vector that can be used to convey genome editing tools, such as CRISPR/Cas9 and base editors; optogenes; or normal genes that require augmentation. In this context, ROs have been utilized to assess various properties of AAV vectors, including their tropism, transduction efficiency, and safety profiles [139–141]. Notably, AAV2 and AAV8, serotypes commonly employed in clinical trials for IRDs, have been specifically evaluated using RO models [142, 143]. Furthermore, AAV5 exhibited greater transduction efficiency in iPSC-derived ROs than AAV9 [27]. However, the limited packaging capacity of AAV vectors (typically less than 5 kb) poses challenges for delivering larger genes. To address this limitation, mRNA trans-splicing dual AAV vectors have been applied to ROs for evaluating the delivery of large genes, including CRISPR/Cas9 and base or prime editors [144]. For genes exceeding AAV capacity, HDAdVs have been employed, as demonstrated in a study using HDAdVs to deliver the 9.4 kb full-length *EYS* gene to ROs, utilizing their 36 kb packaging capacity for long-term transgene expression [94]. Additionally, lipoplexes that can co-encapsulate different mRNAs have been verified using mouse ROs [145].

### Cell Replacement Therapy

Cell replacement therapy is gaining momentum as a promising treatment option for IRDs. Given the limited regenerative capacity of the human retina, damaged retinal cells cannot rejuvenate and are destined for apoptosis [108, 146]; therefore, substituting and supporting injured cells with healthy functional cells is crucial. Immune rejection is the first consideration in cell transplantation, as unmatched transplantation entails various cellular and systemic immune responses. These reactions initiate the death of transplanted cells, which is often followed by the destruction of nearby tissues [81]. This consideration is particularly critical for patients with IRDs who lack a normal retina, which reduces the possibility of successful future transplantation attempts [81]. Additionally, most patients with IRDs have injured retinas with a vulnerable blood-retinal barrier, which destroys their immune-privileged nature and allows the peripheral immune system to recognize these as foreign cells [147]. Collectively, these findings highlight the need to utilize patient-specific iPSCs that have an identical immunological match with the patient for retinal cell transplantation. Moreover, retinal cells in iPSC-ROs possess stratification and cell-to-cell connections that mirror those of the native retina, offering reliability in their function as a source for cell therapy [12].

There are two main approaches to RO-based cell therapy: single-cell suspension and retinal sheet transplantation. Single-cell suspension requires the purification of target cells from ROs through cell-sorting methods, such as fluorescence-activated cell sorting (FACS), magnetic-activated cell sorting (MACS), surface marker panels, and microfluidic enrichment, with the resultant cells mostly being transplanted via subretinal injection [8, 148]. However, certain cell purification methods may compromise cell viability. Retinal sheet transplantation involves transplantation of the entire structure, thus improving graft survival [149, 150]. The retinal sheet also retains apical-basal polarity and lamination, which provides a graft-friendly microenvironment. However, spontaneous rosette formation in a retinal sheet may lead to improper synaptic connections with host cells [151]. ROs can be used in transplantation to amplify specific cells, either in single-cell suspensions or as preorganized tissues [152]. However, controversies regarding the transplantation methods exist, and further research is required to identify the optimal conditions and approaches for utilizing ROs for cell supplementation.

The main targets for cell replacement therapy in IRDs are photoreceptor cells, retinal ganglion cells (RGCs), and the RPE. While the reviewed literature lacks studies on transplanting autologous cells derived from mutation-corrected patient-specific iPSC-ROs, significant efforts have been directed towards expanding the availability of specific cell types for transplantation purposes. Representative studies on cell transplantation therapy are listed in Table 2.

**Table 2:** Representative studies on cell transplantation using human pluripotent stem cell-derived retinal organoids

Cell type	Modality	Cell sorting	Markers for sorting	Preclinical model	Transplantation location	hPSC	References
PRC (PPC)	Sheet	−	−	RD nude rats (*RHO* S334ter)	Subretinal	hiPSC	[95]
	Sheet	−	−	RD nude rats (*RHO* S334ter)	Subretinal	hESC	[153]
	Sheet	−	−	RCS rats	Subretinal	hESC	[154]
	Cell suspension	MACS	CD73	P23H rats (*RHO* P23H)	Subretinal	hiPSC	[155]
	Cell suspension	FACS	L/Mopsin	*rd1* mice (*PDE6β* mutation)	Subretinal	hESC	[156]
						hiPSC	
	Cell suspension	FACS	mCar	*Cpfl1* mice	Subretinal	hiPSC	[157]
			Crx				
	Cell suspension	FACS	RCVRN	−	−	hiPSC	[158]
	Cell suspension	FACS	CD29-/SSEA1-	C57BL/6 wild-type mice, *rd1* mice (*PDE6β* mutation)	Subretinal	hESC	[159]
						hiPSC	
	Cell suspension	FACS	CRX	*rd1* mice (*PDE6β* mutation)	Subretinal	hESC	[160]
	Cell suspension	FACS	SSEA1-/CD133+/CD26+/CD147+	−	−	hiPSC	[161]
	Cell suspension	FACS	Cone-specific L-opsin promoter PR1.7	*rd1* mice	Subretinal	hiPSC	[162]
	Cell suspension	FACS	CRX	C57BL/6J, *Nrl*^*−/−*^, NSG mice	Subretinal	hESC	[163]
	Cell suspension	−	−	13-lined ground squirrel	Subretinal	hiPSC	[164]
	Cell suspension	−	−	*rcd1* dogs (*PDE6β* mutation)	Subretinal	hESC	[165]
	Cell suspension	−	−	*Crb1* KO mice	Subretinal	hiPSC	[147]
	Micro-dissected multilayered retinal fragments	−	−	*rd1* mice (PDE6β mutation)	Subretinal	hESC	[166]
						hiPSC	
RGC	Cell suspension	MACS	Thy1	Optic nerve crush mouse model	Intravitreal	hiPSC	[167]
	Cell suspension	MACS	Thy1.2	Wild-type mice	Intravitreal	hESC	[168]
	Cell suspension	MACS	Thy1.2	Wild-type mice	Subretinal	hESC	[169]
	Cell suspension	FACS	CD184+/CD171+	Optic nerve crush mouse model	Intravitreal	hiPSC	[170]
	Cell suspension	Two-step immunopanning	Thy1	−	−	hiPSC	[171]
RPE	Cell suspension	−	−	RCS rats	Subretinal	hESC	[172]
	Cell suspension	−	−	C57BL/6 wild-type mice, RCS rats	Subretinal	hiPSC	[173]
RPC	Cell suspension	−	−	−	−	hiPSC	[174]
	Cell suspension	FACS	C-Kit+/SSEA4-	RCS rats	Subretinal	hESC	[175]
	Retinal spheroid-derived patch	LF-GC	−	RD nude rats (*RHO* S334ter)	Subretinal	hiPSC	[176]
Müller glia	Cell suspension	FACS	−	Rat model of RGC depletion (NMDA damage)	Intravitreal	hiPSC	[177]

Abbreviations: *PRC* photoreceptor cell, *PPC* photoreceptor precursor cell, *RGC* retinal ganglion cell, *RPE* retinal pigment epithelium, *RPC* retinal progenitor cell, *FACS* fluorescence-activated cell sorting, *MACS* magnetic activated cell sorting, *LF-GC* label-free ghost cytometry, *RCS* royal college of surgeons, *hPSC* human pluripotent stem cell, *hiPSC* human induced pluripotent stem cell, *hESC* human embryonic stem cell

#### Photoreceptor Cells

Photoreceptor cells, that is, rod and cone cells, are frequently disrupted in IRDs such as RP, LCA, and Stargardt disease. Recent advancements in photoreceptor transplantation have shown promising results in preclinical studies.

Photoreceptor cell sheets derived from hESC-ROs have been successfully engrafted onto Royal College of Surgeons (RCS) rats, demonstrating functional integration with host retinal cells [154]. Furthermore, single-cell suspensions of human photoreceptor cells obtained from ROs have been transplanted into murine models of retinal degeneration, forming synapses with host cells and eliciting light responses [155–157]. Cell purification from ROs has been achieved using cell-surface marker panels or photoreceptor cell reporter human iPSC (hiPSC) lines [158, 159].

Several studies have focused on enriching specific photoreceptor subtypes, particularly cone cells, by enhancing cone signals using cone-specific markers and subsequently isolating these cells using FACS [160, 161]. Similarly, cone photoreceptors from hiPSC-ROs have been manipulated to express microbial opsins and fluorescent reporter genes, facilitating subsequent isolation [162]. Upon transplantation into *rd1* mice, these cone cells exhibited heightened sensitivity to light, potentially improving temporal resolution. Efforts to increase photoreceptor yields have employed various methods, including the utilization of bioreactors, ultimately yielding an ample supply of mature photoreceptors [178].

Subretinal transplantation of photoreceptor precursor cells derived from hPSC-ROs into animal models of retinal degeneration has shown success [147, 165]. A notable study demonstrated that hESC-derived RO sheets, even after overnight shipping, could successfully integrate into host retinas of RD nude rats, forming functional photoreceptors and improving vision [153]. Importantly, the response heatmap in the superior colliculus corresponded to the transplant location in the host retina, suggesting point-to-point projection. Recent investigations into photoreceptor transplantation have revealed species-specific limitations in material transfer between transplanted and host cells, emphasizing the importance of considering species compatibility in developing effective transplantation strategies [163]. A novel study using 13-lined ground squirrels as a model for cone-rich retinal regions has shown successful long-term survival of transplanted hiPSC-derived photoreceptors [164]. This research employed advanced imaging techniques, including adaptive optics scanning light ophthalmoscopy, to non-invasively monitor transplanted cells for up to four months. This model offers new opportunities for developing cone replacement therapies, addressing a gap in commonly used rodent models that lack cone-rich regions.

Watari et al. devised a quality control method for hiPSC-derived retinal sheets for subretinal transplantation [95]. The authors dissected the neuroepithelium into an inner “cap” for transplantation and an outer “ring” for qPCR analysis, effectively excluding corresponding off-target tissues. While the peripheral “ring” tissues were used for qPCR, a non-freezing preservation method was used to successfully store the retinal sheet at 17 ± 5 °C in Optisol-GS for up to 4 days. Subsequent transplantation of these sheets into rats with retinal degeneration resulted in the long-term survival of mature and functional photoreceptors.

A recent groundbreaking clinical trial (jRCTa050200027) has demonstrated the long-term safety and stability of allogeneic iPSC-derived RO sheet transplantation in patients with advanced RP [179]. In this first-in-human study involving ROs, RO sheets were successfully transplanted into two subjects, with the grafts surviving without immune-mediated rejection or tumor formation for two years. The transplantation procedure increased retinal thickness at the graft site without serious adverse events, and changes in visual function were less pronounced in the treated eye compared to the untreated eye. This study provides evidence for the potential of iPSC-derived RO transplantation as a therapeutic approach for retinal degeneration, paving the way for further investigations into its safety and efficacy for visual function restoration.

#### Retinal Ganglion Cells

Retinal ganglion cells (RGCs) are commonly injured in glaucoma, optic neuritis, and Leber’s hereditary optic neuropathy. Considering that substantial RGC loss occurs prior to the diagnosis of blindness related to RGC degeneration, cell transplantation has emerged as a potential treatment option [180].

Recent studies have demonstrated the successful generation, purification, and transplantation of RGCs derived from hESC-ROs. These transplanted cells have shown survival and integration in the host retina for up to one-week post-transplantation, highlighting the potential of this approach for RGC replacement therapies [168, 170]. RGCs generated and purified from ROs expressed RGC markers, with those harvested at day 90 exhibiting optimal survival and neurite extension [171]. Upon transplantation into mice with optic neuropathy, these isolated RGCs have shown the capacity to survive for more than one-month post-treatment and incorporate into the host ganglion cell layer [167].

A novel technique employing subretinal injection combined with chemokine-induced migration has shown promise in enhancing RGC integration. This method resulted in a 2.7-fold increase in RGC migration into the ganglion cell layer and retinal nerve fiber layer, with migrated cells expressing mature RGC markers. This technique addresses the limitations associated with intravitreal injections, where donor neurons often fail to integrate beyond the internal limiting membrane [169].

For successful RGC replacement, axon regeneration and the establishment of appropriate synaptic connections to the brain are crucial [181]. However, these processes are challenging because axons need to stretch several centimeters to extend into the brain while simultaneously establishing specific rewiring of targets [181, 182]. Alternative approaches include supporting endogenous RGCs with Müller glial or neural progenitor cells derived from ROs [96]. Notably, Müller glial cells isolated from ROs have demonstrated the ability to enhance RGC function and visual response [177].

#### Retinal Pigment Epithelium

The RPE is commonly damaged in IRDs such as RP and Stargardt disease. While RPE cells can be generated directly from hPSCs, they can also be obtained concurrently with ROs as adjacent structures to the optic cup. Historically, direct induction methods have been favored for RPE differentiation from PSCs. However, recent advancements have facilitated the generation of functionally mature, polarized RPE monolayers from iPSC-derived ROs. This novel approach involves isolating RPE spheroids from the NR component of organoids, followed by enzymatic dissociation and seeding onto Matrigel-coated transwell filters. Under these conditions, the dissociated RPE cells exhibit behavior similar to primary human RPE cells, forming characteristic pigmented cobblestone monolayers [183].

The potential of RO-derived RPE for transplantation has been demonstrated in animal models. For instance, transplantation of hESC-derived 3D RPE cells into RCS rats proved safe and effectively rescued retinal degeneration, demonstrating the potential of ROs for RPE transplantation [172]. A meta-analysis of RPE transplantation in animal models of retinal degeneration revealed significant improvements in electroretinogram responses and vision-based behaviors [184]. Efforts to enhance RPE transplantation efficacy have led to innovative approaches, such as using acellularized uveal hydrogel as a vehicle for subretinal RPE cell injection. This method has shown promising results in RCS rats, enhancing cell integration, reducing cell death and retinal gliosis, and restoring long-term vision [173].

Translating these preclinical findings to clinical applications, several clinical trials have been conducted using hESC- and hiPSC-derived RPE cells, transplanted either as sheets or cell suspensions. These studies have demonstrated short-term safety and improvements in visual function for patients with advanced-stage Stargardt disease and age-related macular degeneration [185–187].

#### Retinal Progenitor Cells

Retinal progenitor cells (RPCs) represent a promising source for cell-based therapies in retinal degeneration. Various approaches have been developed to isolate RPCs from ROs. One method involves generating RPC lines from ROs using fluorescent reporter iPSC lines [174]. When RPCs isolated from hESC-derived ROs were transplanted subretinally into RCS rats, improvements in visual function and outer nuclear layer thickness were observed, along with the promotion of Müller glia dedifferentiation [175]. A recent advancement in RPC isolation is the label-free ghost cytometry (LF-GC) approach. This machine learning-optimized method efficiently produces transplantable retinal spheroids without requiring specific surface antigens or fluorescent labeling. LF-GC-sorted RPCs have demonstrated high graft indices and potential for structural maturation and synaptic integration in vivo [176]. This technique represents a significant step towards streamlining the preparation of clinical-grade grafts for retinal cell therapies, potentially enhancing the feasibility and efficacy of treatments for IRDs.

For clinical translation of hPSC-derived retinal cells, good manufacturing practice (cGMP)-compliant protocols should be developed to ensure immunological safety and meet the regulations of federal agencies [81]. Xeno-free and feeder-free culture methods have substituted undefined conditions of non-human derivatives; for example, FBS and Matrigel were replaced with human platelet lysate and recombinant laminin, respectively [37, 188]. Wiley et al. developed a cGMP-compliant RO differentiation protocol that utilizes a cGMP facility throughout the derivation of ROs [81]. Further research is necessary to develop an efficient and optimized cGMP-compliant protocol for the clinical translation of ROs. To serve as a potential option for a definitive cure for IRDs, optimization of transplantation conditions, including the transplantation site, method, and transplanted cell type, should precede clinical translation.

### Limitations and Potential Applications

RO technology has progressed significantly over the last decade. ROs have been applied in various areas of vision research, providing valuable insights and yielding new developments. However, several challenges in improving RO technology remain and must be addressed.

While patient-derived ROs offer promising opportunities for personalized medicine, we acknowledge the challenges in feasibility and cost-effectiveness. Not all patient iPSC lines generate ROs with equal efficiency, and the process can be resource-intensive [8]. Recent advancements in standardizing RO generation protocols have improved consistency, but variability between different iPSC lines remains a concern [189].

To address these challenges in personalized medicine applications, alternative approaches are being explored. One strategy, which aims to balance personalization with scalability, is the use of HLA-matched allogeneic iPSCs. For example, a clinical trial has been conducted involving the use of RPE cells derived from HLA-matched allogeneic iPSCs for macular degeneration [190]. This approach may offer a more scalable solution while potentially reducing the need for immunosuppression compared to fully allogeneic transplants. Despite these challenges, patient-derived ROs remain valuable, particularly for rare genetic variants where standard treatments are ineffective.

Morphological advancements in RO technology are required to overcome current limitations. While ROs include all major retinal cell types and form 3D laminar structures, they lack connections of RGCs to the brain and other important cellular components such as endothelial cells and microglia. Spontaneous differentiation from the neuroectoderm does not yield structures other than the NR or RPE; therefore, additional manipulations are required to introduce cells of various lineages, especially mesodermal cells related to vascular and immune systems. During long-term culture, the inner retinal layers in some organoid models may be compromised or reduced due to the absence of a vascular supply and synaptic connections to the brain. Current RO protocols depend on the free diffusion of oxygen and nutrients; therefore, introducing vasculature could potentially enhance the efficient differentiation of retinal cells [191]. To address these limitations, various research groups have introduced vasculature into neural organoids using co-culture, 3D bioprinting, microfluidics, assembloids, and implantation [191]. Building on these advancements, several studies have reported the development of retinal-brain assembloids and vascularized neural organoids [192–195].

Additionally, in current organoid technology, ROs merely retain the RPE without connecting to the photoreceptor outer segments. Contact between the outer segment and RPE is necessary for the daily shedding and renewal of outer segment membrane disks [196]. The RPE participates in the visual cycle by phagocytosing outer segment membrane disks and recycling retinoic acid. Ions, metabolites, and fluids are transported between the RPE and photoreceptor outer segments [197]; therefore, establishing a close connection is imperative for achieving a more accurate understanding of the microenvironment. Organ-on-a-chip technology and co-culture conditions that promote the connection of the RPE to the NR are strategies to compensate for the deficits of current organoid technology [198].

Furthermore, minimizing manual labor while simultaneously increasing efficiency and reducing heterogeneity is essential to ensure the universal use of RO technology. Platforms have been developed to circumvent the time-consuming manual dissection procedure using a 3D agarose micromold or agarose microwell array seeding and scraping [188, 199]. Further, hydrogel-based milliwell arrays have been employed to bypass manual procedures for standardized and expandable generation of ROs [200]. Advancements in culture technology involving the retina-on-a-chip, biomaterial scaffolds, and bioreactors have enhanced culture yields [17, 178, 201]. The prediction of RO differentiation using technologies such as convolutional neural networks is considered a preliminary step in the development of next-generation RO technologies [202, 203].

**Fig. 6:**
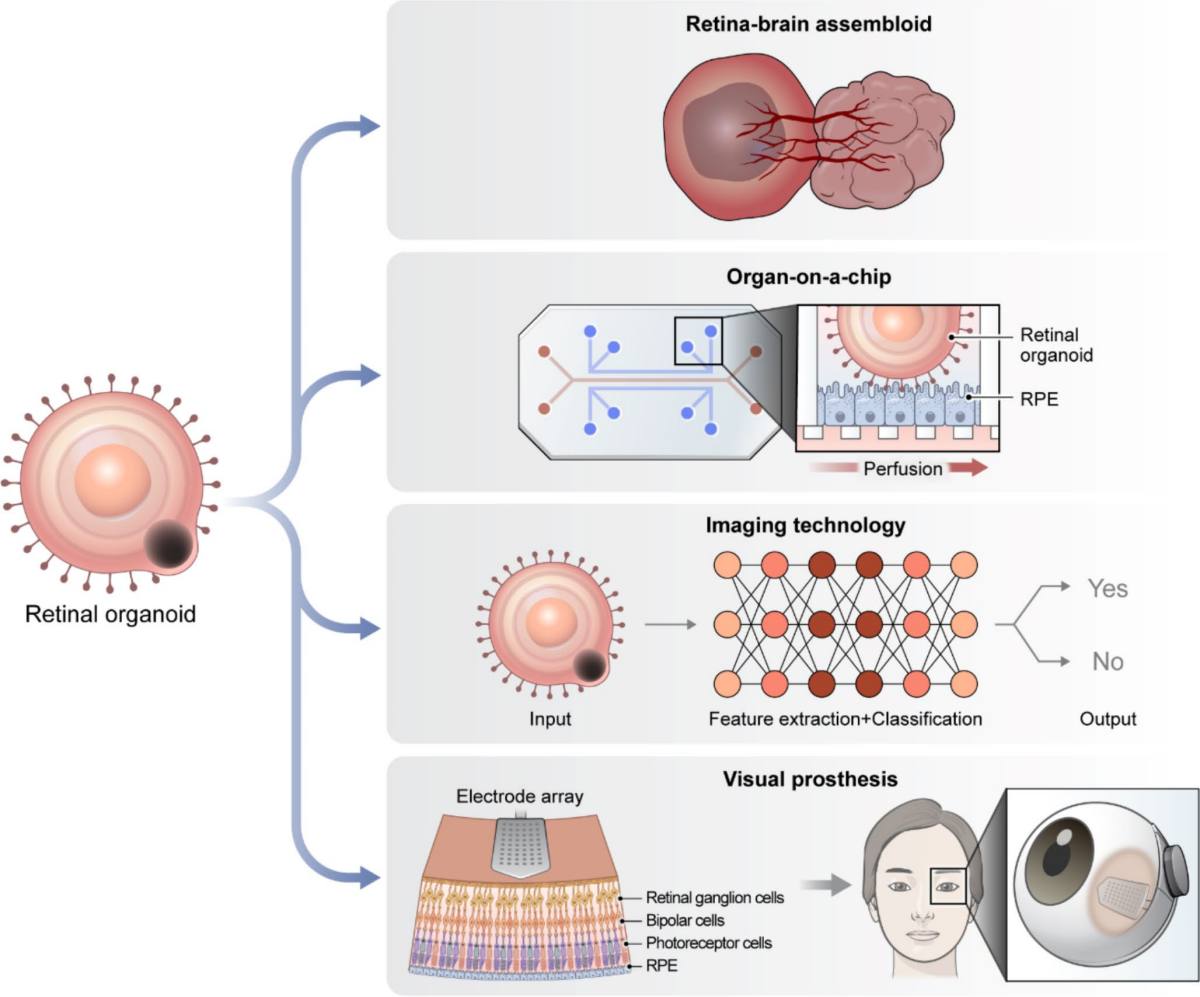
Potential applications and advancements in retinal organoid technology. Retinal organoid culture systems can be improved by assembloid technology and organ-on-a-chip approaches. In addition, retinal organoid technology can be combined with advanced imaging technology and visual processing research for further applications. These combined technologies offer improved models for studying retinal development, disease mechanisms, and potential therapeutic strategies. Abbreviation: RPE, retinal pigment epithelium

While these limitations present ongoing challenges, they also drive innovation and open up new avenues for research and application. As we continue to address these challenges, the potential applications of RO technology continue to expand, offering promising opportunities across various fields of study and clinical practice (Fig. 6).

The use of ROs could be expanded to include various diseases and organs. Retinal manifestations of specific genetic or systemic diseases can be investigated using RO technology. Considering that the retina serves as a continuation of the central nervous system within the eye, phenotypes associated with neurological disorders can be explored using ROs [204, 205]. For instance, ROs can be generated from the cells of patients with genetic mutations related to diseases such as familial Alzheimer’s disease [206]. Furthermore, interactions between the retina and the brain can be evaluated by co-culturing or forming assembloids of these two components. Indeed, RGC axon projections to the brain have been recapitulated using retinal-brain assembloid technology [192]. Additionally, RO technology has been used to assess systemic toxicity in the retina [207]; this approach could be extended to organs other than the eye.

Advanced imaging technologies and image processing have been integrated into RO technology to provide nondestructive quality control tests [208]. Precise and non-invasive methods are required to ascertain the functionality and safety of ROs, owing to their intricate structures. The application of computational image-processing technologies, including deep learning technology, could sort ROs without compromising integrity, thus improving both the accuracy and efficiency of image analysis. Additionally, imaging technology could aid in the standardized assessment of ROs, thus minimizing the variability in RO production.

RO technology provides a wide variety of treatment options as a platform for vision research; however, to date, studies on light-evoked responses to ROs are limited [15]. Whole-cell/perforated patch clamp experiments or microelectrode arrays can not only help identify the electrophysiological properties of ROs but also yield functional, light-responsive ROs [209]. This approach can lead to the development of various electronic visual support devices and prostheses [210]; however, pre-transplantation experiments are essential before RO technology can be extended to patients.

## Conclusions

The preparation of ROs is a complicated process involving accurate design and concentration. Current preparation protocols require modifications to provide a robust foundation for advancing vision research. The illustrated cases of ROs generated from iPSCs derived from patients with IRDs demonstrate the potential of this technology to precisely recapitulate disease phenotypes and act as promising disease models for research on pathogenesis. The therapeutic techniques explored using RO technology provide a platform for introducing new interventions into IRD research. Further exploration of the intricacies of RO technology would reveal the promising potential this versatile tool holds for both ocular research and clinical applications.

## Data Availability

Not applicable.

## Abbreviations

2D: Two-dimensional
3D: Three-dimensional
AAV: Adeno-associated virus
ASO: Antisense oligonucleotide
BMP4: Bone morphogenetic protein 4
cGMP: Current good manufacturing process
CNV: Copy number variation
ESC: Embryonic stem cell
FACS: Fluorescent-activated cell sorting
FBS: Fetal bovine serum
HDAdV: Helper-dependent adenoviral vector
hESC: Human embryonic stem cell
hiPSC: Human induced pluripotent stem cell
hPSC: Human pluripotent stem cell
iPSC: Induced pluripotent stem cell
IRD: Inherited retinal disease
LCA: Leber congenital amaurosis
LF-GC: Label-free ghost cytometry
MACS: Magnetic-activated cell sorting
NR: Neural retina
PRISMA: Preferred reporting items for systematic reviews and meta-analyses
PSC: Pluripotent stem cell
RA: Retinoic acid
RB: Retinoblastoma
RCS: Royal College of Surgeons
RGC: Retinal ganglion cell
RO: Retinal organoid
RP: Retinitis pigmentosa
RPC: Retinal progenitor cell
RPE: Retinal pigment epithelium
SAG: Smoothened agonist
scRNA-seq: single-cell RNA sequencing
SFEBq: Serum-free culture of embryoid body-like aggregates with quick reaggregation
TRID: Translational readthrough-inducing drug
XLRS: X-linked retinoschisis
